# Comparable Effects of Strontium Ranelate and Alendronate Treatment on Fracture Reduction in a Mouse Model of Osteogenesis Imperfecta

**DOI:** 10.1155/2021/4243105

**Published:** 2021-01-08

**Authors:** Changgui Shi, Bin Sun, Chao Ma, Huiqiao Wu, Rui Chen, Hailong He, Ying Zhang

**Affiliations:** ^1^Department of Orthopedics, Changzheng Hospital, Second Military Medical University, Shanghai, China; ^2^Department of Plastic and Reconstruction Surgery, The General Hospital of Chinese People's Liberation Army, Beijing, China

## Abstract

Alendronate (Aln) has been the first-line drug for osteogenesis imperfecta (OI), while the comparable efficacy of Aln and strontium ranelate (SrR) remains unclear. This study is aimed at comparing the effects of SrR and Aln treatment in a mouse model of OI. Three-week-old oim/oim and wt/wt female mice were treated with SrR (1800 mg/kg/day), Aln (0.21 mg/kg/week), or vehicle (Veh) for 11 weeks. After the treatment, the average number of fractures sustained per mouse was significantly reduced in both SrR- and Aln-treated oim/oim mice. The effect was comparable between these two agents. Both SrR and Aln significantly increased trabecular bone mineral density, bone histomorphometric parameters (bone volume, trabecular number, and cortical thickness and area), and biomechanical parameters (maximum load and stiffness) as compared with the Veh group. Both treatments reduced bone resorption parameters, with Aln demonstrating a stronger inhibitory effect than SrR. In contrast to its inhibitory effect on bone resorption, SrR maintained bone formation. Aln, however, also suppressed bone formation coupled with an inhibitory effect on bone resorption. The results of this study indicate that SrR has comparable effects with Aln on reducing fractures and improving bone mass and strength. In clinical practice, SrR may be considered an option for patients with OI when other medications are not suitable or have evident contraindications.

## 1. Introduction

Osteogenesis imperfecta (OI) is a genetic disorder in connective tissues characterized by increased bone fragility. The clinical manifestations of OI range widely from fractures to skeletal deformities, and even death [1]. Frequent fractures have been the hallmark of OI, and the risk of fractures is highest in childhood and adolescent years [2], which may bring huge physical, psychological, and economic burdens.

The goal of OI treatment varies with phenotype and mobility status. To date, there has been no curative treatment for OI. Bisphosphonate (BP), which inhibits osteoclast-mediated bone resorption by inhibiting farnesyl diphosphate synthetase, has been the standard treatment for OI and demonstrated effectiveness in increasing bone mineral density (BMD), reducing pain, and decreasing fracture [3–5]. However, this type of medication may result in undesirable outcomes, such as impairment in bone healing and modeling, damage to bone cells, and accumulation of microdamage in the bone [6, 7]. Due to the long half-life of BP, its use in children warrants a closer examination, especially when used for young girls who usually reach reproductive maturity within a decade of treatment. Additionally, the optimal duration of BP treatment remains unclear [8, 9]. Therefore, an increasing number of studies have searched for other promising agents such as strontium ranelate (SrR) [10].

Strontium ranelate is an extensively studied medication mainly used for postmenopausal osteoporosis to decrease fractures in vertebral and nonvertebral bones [11–13]. SrR consists of two atoms of stable strontium (Sr^2+^) and an organic part. Sr^2+^ is a bone-seeking trace element which can partially substitute Ca^2+^ in the hydroxyapatite crystal lattice and thus be incorporated into bone mineral [14]. As an effective component of strontium ranelate, Sr^2+^ could decrease bone resorption while maintaining bone formation via activating calcium-sensitive receptor pathway [15–17]. Geoffroy and colleagues [18] found that SrR decreased the caudal vertebral fracture in a mouse model with spontaneous fractures. SrR also significantly reduced fracture incidence in a growing mouse model of OI [10], which may be explained by the positive effect of SrR on bone mass and strength. Despite the increasing number of studies investigating SrR, little is known about the comparative effects of SrR and the traditionally used BP in the treatment of OI.

Based on the above evidence, the aim of this study was to compare the effects of SrR and alendronate (Aln) treatment using the oim/oim mouse model. The oim/oim mouse has been a frequently used animal model for moderate to severe type III OI [19]. This model has a naturally occurring mutation causing the deficiency of pro-*α* 2(I) collagen, which can result in various phenotypic and biochemical features, such as limb deformities, frequent fractures, osteopenia, and small size [19]. These features are very similar to those seen in human OI. This model has been successfully used in previous studies that evaluated the effect of BP or SrR in OI treatment. In this study, we hypothesized that SrR has comparable effects with Aln on reducing fractures and improving bone mass and strength.

## 2. Materials and Methods

### 2.1. Animals and Treatment

All animal procedures were approved by the Animal Care Committee at the Second Military Medical University. All applicable institutional and/or national guidelines for the care and use of animals were followed. Detailed descriptions of the animals and study procedures were reported previously [10]. Briefly, a group of homozygous oim/oim (*n* = 36) and wild-type (wt/wt) (*n* = 36) female mice were used. These mice were bred from heterozygous B6C3Fe-a/a-Colla2^oim/+^ hybrid oim/wt breeder animals from the Jackson Laboratories (BarHarbor, ME, USA) and were genotyped using polymerase chain reaction following the Saban and King protocol [20]. The mice were housed in cages containing four mice according to genotype in a light-controlled environment. They were fed with tap water and powdered rodent diet and weaned at 3 weeks of age [21].

The mice were assigned randomly to one of the six groups (*n* = 12 each) based on the genotype (oim/oim and wt/wt) and treatment (vehicle (Veh), SrR, and Aln). Starting at 3 weeks of age, mice in the SrR groups were treated with SrR (1800 mg/kg/day, gavage), and mice in the Aln groups were administrated (0.21 mg/kg/week, subcutaneously injection). These dosages were chosen based on previous reports [18, 22, 23]. Mice in the Veh groups were treated with vehicles (1% hydroxyethylcellulose aqueous solution by gavage and saline by subcutaneous injection) from 3 to 14 weeks (skeletal maturity) [24, 25] as control groups. The oim/oim and wt/wt genotypes received the same treatments for 11 weeks. All mice were weighed weekly to adjust the dosage and track weight change. Five mice were excluded from the analysis due to accidental death (2 from oim/SrR group, 2 from oim/Aln group, and 1 from oim/Veh group). Ten and three days prior to euthanasia, all mice were administered tetracycline at 30 mg/kg (Sigma-Aldrich, St. Louis, MO, USA) for the preparation of dynamic histomorphometry.

### 2.2. Radiographic Analysis

At the end of the treatment, Faxitron X-ray (Wheeling, PA, USA) was used to obtain whole-body high-resolution radiographs. Two independent investigators were blinded and counted fractures in the femurs, tibiae, humeri, radii, and individual tail bones. Any evidence of callus formation or obvious bone deformation was considered a fracture. The total number of fractures was obtained for each mouse. Radiographs for isolated femurs were also obtained at the anterior-posterior (AP) and medial-lateral (ML) planes (resolution: 20 linear pixels per millimeter). The femoral length was defined as the distance from the top of the femoral head to the bottom of the condyles. The endosteal (de) and periosteal (dp) diameters were measured at the midpoint of the femur. The moment of inertia (I) was calculated with the following formula:

(1) $$\begin{array}{c} I=\frac{\pi }{64}\left[{\left(\text{APdp}\right)\left(\text{MLdp}\right)}^{3}-{\left(\text{APde}\right)\left(\text{MLde}\right)}^{3}\right], \end{array}$$

where APdp corresponds to the major diameter of the ellipse and MLdp plane corresponds to the minor diameter [10]. In this study, moments of inertia were assessed using Faxitron, and the femur was assumed to be elliptical, in line with previous studies [22, 26, 27].

### 2.3. Serum Biochemistry

At the end of the treatment, blood samples were collected via intracardiac puncture. The serum was obtained by centrifuging the blood samples and stored at –80°C until being analyzed. The ELISA kit (BT-470; BTI, Stoughton, MA, USA) was used to quantify serum osteocalcin (OCN) as a measure of osteoblast activity. Serum cross-linked N-telopeptide of type I collagen (NTx) (Mouse NTx; Uscn Life Science & Technology Company, Wuhan, China) was assessed as a measure of osteoclast resorption. The serum tests were performed in duplicates.

### 2.4. Micro-Computed Tomography (Micro-CT)

At sacrifice, the left femur of each mouse was obtained and tested by Micro-CT (Skyscan1176; Bruker, Kontich, Belgium) using a 9 *μ*m voxel size, 50 kVp, 500 mA, and 0.7 rotation step. The Feldkamp algorithm within the cone-beam reconstruction software (version 1.13.11.0) was used for 3D reconstruction and data analysis. The regions of interest (ROIs) at trabecula were defined as 90 *μ*m area from 50 *μ*m proximally to the end of the distal growth plate toward the direction of femoral diaphysis. The ROIs at cortex were defined as 90 *μ*m slices at the middle femoral diaphysis. The following properties were identified: the moments of inertia, trabecular BMD, bone volume fraction (BVF), trabecular number (Tb.N), trabecular thickness (Tb.Th), trabecular separation (Tb.Sp), cortical BMD, cortical thickness (Cort.Th), cortical cross-sectional area (Cort.CSA), total area, marrow area, and periosteal perimeter [28].

### 2.5. Dynamic Bone Formation

At sacrifice, the left tibia of each mouse was separated and used to evaluate the dynamic bone formation. The samples were processed according to the previous study [10]. After dehydrating by the graded series of ethanol (70% to 100%), the bones were embedded in methyl-methacrylate (MMA; Sigma-Aldrich, St. Louis, MO, USA) with 10% dibutyl phthalate and 0.05% benzoyl peroxide. The samples were the cut transversally at the middle femoral diaphysis with a microtome (Leica RM2255, Leica Biosystems Inc., Buffalo Grove, IL, USA). The proximal parts were used to cut and polished to several slices at an approximately 5 mm thickness. The slices were then imaged via a fluorescent microscope (Zeiss Axioplan microscope, Thornwood, NY, USA). The measurements included single-labeled surface (sLS), double-labeled surface (dLS), and interlabel thickness (IrLTh). The mineral apposition rate (MAR = Ir.L.Th/7 days; *μ*m/day), mineralizing surface/bone surface ratio (MS/BS = [1/2 sLS + dLS]/BS; %), and bone formation rate (BFR = MS/BS × MAR; *μ*m^3^/*μ*m^2^/day) were then calculated according to the guidelines by the American Society for Bone and Mineral Research [29]. Image-Pro Plus (Media Cybernetics, Rockville, MD, USA) was used for calculation.

### 2.6. Histology

At sacrifice, the right tibia was dissected and fixed in 4% paraformaldehyde. After decalcification, the bones were embedded in paraffin and cut longitudinally at 4 mm thickness section. The tartrate-resistant acid phosphatase (TRAP) (#387A-1KT; Sigma-Aldrich, St. Louis, MO, USA) was used to stain the osteoclasts. The number of osteoclasts (N.Oc/BS) and the osteoclast surface (Oc.S/BS) were measured at the primary spongiosa in a 0.5 × 0.5 mm^2^ region from two separate sections. Other sections were used for immunohistochemical staining, according to standard methods [10]. After deparaffinization, the bone sections were incubated in 3% hydrogen peroxide for 15 minutes and then blocked in 3% normal goat serum for 30 minutes. The sections were then incubated overnight with primary antibodies to rabbit anti-mouse Type I collagen (1 : 500 dilution, Abcam, Cambridge, MA, USA), followed by incubation using a goat anti-rabbit biotin-labeled secondary antibody. After washing with phosphate buffer saline, sections were stained with peroxidase-labeled streptavidin–biotin technique (DAB kit, Invitrogen) and counterstained with hematoxylin. The sections were imaged via a light microscopy at ×40 magnification at the primary spongiosa. Image-Pro Plus (Media Cybernetics, Rockville, MD, USA) was used for the histology and immunohistochemistry analysis.

### 2.7. Biomechanical Testing

The right femur of each mouse was used to perform the three-point bending tests (Model 5569; Instron Corp., Norwood, MA, USA) according to previous description [10]. Only femurs with no fractures or obvious deformities on the radiographs were used for testing. During the bending testing, each femur was centered on two supports spaced 6 mm apart (L) with the anterior cortex placing in longitudinal compression and the posterior cortex in tension. The load was applied at a constant displacement rate of 1 mm/min at the midpoint until the bone fractured. The choice of 6 mm support span was based on previous studies [30, 31]. The following structural mechanical properties were identified and calculated: the maximum load, stiffness, energy to failure, yield displacement, and postyield displacement. The inherent material properties were then calculated by the load and displacement data which were normalized to the moment of inertia. Stress was calculated as *σ* = *APdpFL*/8*I* (*F* is force during loading). Strain was calculated as *ε* = 6*APdpD*/*L*^2^ (*D* is the displacement during loading). Young's modulus was calculated as the slope of the linear ascending region of the stress-strain curve.

### 2.8. Statistical Analysis

Statistical analyses were performed using SPSS version 18 (SPSS Inc., Chicago, IL, USA). Data were presented as mean ± SD. Comparisons were made between three different treatments (e.g., vehicle, SrR, and Aln), as well as oim/oim and wt/wt animals. For micro-CT, bone formation, and histology assays, only the femur with no fractures or deformities at the middle and distal end was selected for analysis. The number of mice from the wt/wt group was matched to their oim/oim littermates for analysis. Two-way ANOVA was used to examine the additive effects of genotype (oim, wt) and treatment (SrR, Aln, and Veh). If significant differences were found for any factors, multiple comparisons were performed using the Student-Newman-Keuls (SNK) or Dunnett test to account for differences among SrR, Aln, and Veh treatments within either oim/oim mice or wt/wt mice. For fracture quantification, a nonparametric measurement, the Mann–Whitney test was used. Statistical significance was set at *p* < 0.05 (two-tailed).

## 3. Results

### 3.1. Growth

**Figure 1:**
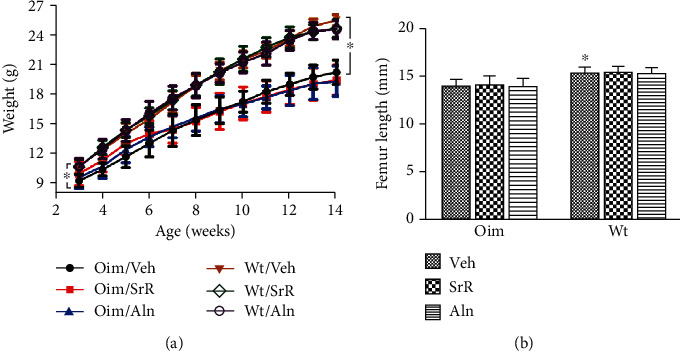
Evaluation of the animal growth for the duration of the study. (a) Growth curves of the bodyweight for different groups of mice. Veh-treated oim/oim mice remained significantly smaller than their wt/wt mice at both 3 and 14 weeks. SrR or Aln treatment did not change the body weight of either genotype. (b) Femoral length of the animals from different groups at 14 weeks of age. The oim/Veh mice had shorter femoral length than wt/Veh mice. SrR and Aln had no effects on femoral length. Values were expressed as mean ± SD; *n* = 11 for oim/Veh group (missing data due to death), *n* = 10 for oim/SrR and oim/Aln group (missing data due to death), *n* = 12 for all the wt groups. ∗*p* < 0.05 wt/Veh vs. oim/Veh.

Analysis of mouse body weight revealed that the Veh-treated oim/oim mice were significantly smaller than the Veh-treated wt/wt mice at both 3 and 14 weeks (Figure 1(a)). There were no significant differences in weight gain among the Veh, SrR, or Aln groups for either wt/wt or oim/oim genotype for the duration of the study (Figure 1(a)). At the end of the treatment, the oim/oim mice had shorter femur lengths than their wt/wt counterparts. SrR and Aln had no effects on femoral length in the oim/oim mice or wt/wt mice after 11 weeks of treatment (Figure 1(b)).

### 3.2. Fracture Analysis

**Figure 2:**
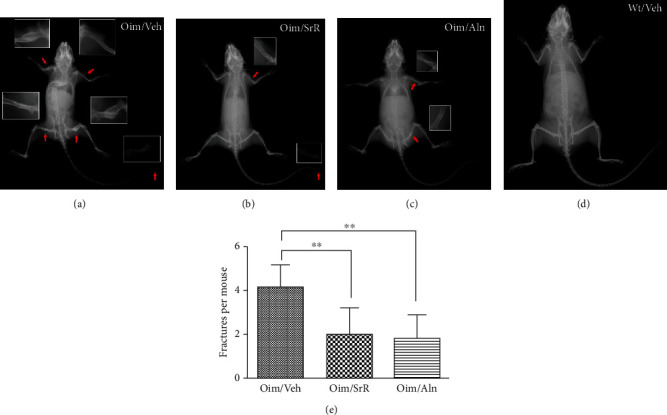
Effects of different treatments on fracture reduction in oim/oim mice. Representative radiographs of (a) Veh-treated, (b) SrR-treated, and (c) Aln-treated oim/oim mice. Red arrow: fractures at different locations. (d) Representative radiograph of Veh-treated wt/wt mice. (e) Statistical analysis of fractures sustained at 14 weeks of age by oim/oim mice after 11 weeks of treatment with Veh, SrR, and Aln. Compared with Veh treatment, the number of fractures was significantly reduced with SrR or Aln treatment. Values were expressed as mean ± SD; *n* = 11 for the oim/Veh group (missing data due to death), *n* = 10 for the oim/SrR and oim/Aln group (missing data due to death). ∗∗*p* < 0.01 compared to oim/Veh.

No fractures were identified in any of the wt/wt mice (Figure 2(d)). Among the oim/oim mice, the average number of fractures sustained per mouse was significantly lower in the SrR and Aln group than in the Veh group at the end of the treatment; the Veh group sustained 4.2 ± 1.0 fractures per mouse, and the SrR- and Aln-treated mice had 2.0 ± 1.2 and 1.8 ± 1.1 fractures per mouse, respectively (Figures 2(a)–2(c) and 2(e)). There was no significant difference between SrR and Aln groups. There were no differences in the locations of fractures in any one of these three groups.

### 3.3. Bone Turnover Markers

**Figure 3:**
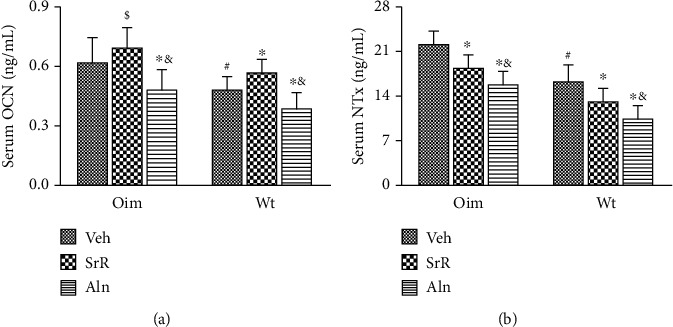
Effects of different treatments on serum bone metabolism markers. oim/Veh mice had higher serum levels of (a) OCN and (b) NTx than wt/Veh mice, suggesting increased bone turnover in oim/oim mice. Compared with Veh treatment, SrR treatment significantly increased serum (a) OCN level in wt/wt mice and decreased (b) NTx level in both oim/oim and wt/wt mice. Aln treatment significantly decreased serum levels of (a) OCN and (b) NTx in either genotype as compared with Veh treatment. Serum NTx level was reduced much more with Aln treatment than with SrR treatment in both genotypes. Values were expressed as mean ± SD; *n* = 10 animals per group (missing data due to death or inadequate blood sample). ∗*p* < 0.05, compared with the same genotype Veh treatment. ^#^*p* < 0.05, wt/Veh vs. oim/Veh. ^&^*p* < 0.05, SrR vs. Aln for the same genotype. ^$^*p* < 0.05, wt/Veh vs. oim/SrR group or oim/Aln group.

Serum markers of bone metabolism, OCN and NTx, were significantly higher in the Veh-treated oim/oim mice than in the Veh-treated wt/wt mice, consistent with reported increases in OI bone turnover [10, 32]. As compared with Veh treatment, SrR treatment significantly increased serum OCN level in wt/wt mice (+19%) but not in oim/oim mice (Figure 3(a)). The serum NTx level was significantly decreased in both oim/oim (-18%) and wt/wt (-20%) mice after SrR treatment (Figure 3(b)). In contrast, 11 weeks of Aln treatment significantly decreased both the serum OCN (-23% oim; -19% wt) and NTx (-28% oim; -35% wt) levels in either genotype as compared with Veh treatment (Figures 3(a) and 3(b)). Furthermore, the serum NTx level with Aln treatment was reduced much more than the SrR treatment in both genotypes (Figure 3(b)).

### 3.4. Bone Mass and Architecture

**Figure 4:**
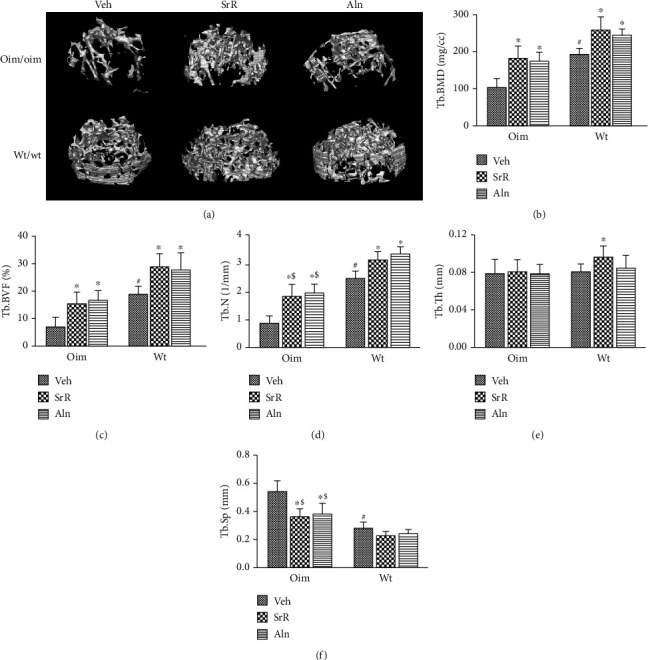
Effects of different treatments on femora trabecular bone mass. (a) Representative micro-CT images of trabecular regions in femora reveal positive effects on bone mass with SrR or Aln treatment. (b–d) Tb.BMD, Tb.BVF, and Tb.N were significantly increased with SrR or Aln in both wt/wt and oim/oim mice. (e) Tb.Th was significantly increased with SrR in wt/wt mice. (f) Tb.Sp was significantly decreased with SrR or Aln in oim/oim mice. Values were expressed as mean ± SD; *n* = 6 animals per group for both oim/oim and wt/wt groups (missing data due to deformity or fractures). ∗*p* < 0.05, compared with the same genotype vehicle. ^#^*p* < 0.05, wt/Veh vs. oim/Veh. ^&^*p* < 0.05, SrR vs. Aln treatment for the same genotype. ^$^*p* < 0.05, wt/Veh vs. oim/SrR group or oim/Aln group.

Structural parameters of the trabecular and cortical bone of the femora are shown in Figure 4. The oim/Veh mice had significantly lower trabecular BMD, BVF, and Tb.N, and higher Tb.Sp as compared with wt/Veh mice. SrR or Aln treatment significantly increased trabecular BMD (+78% oim and +34% wt in SrR; +70% oim and +27% wt in Aln), BVF (+119% oim and +53% wt in SrR; +137% oim and +47% wt in Aln), and Tb.N (+106% oim and +26% wt in SrR; +123% oim and +34% wt in Aln) for both genotypes (Figures 4(b)–4(d)). Both SrR- and Aln-treated oim groups showed increases in Tb.BMD and BVF to levels not significantly different from the vehicle-treated wt/wt mice (Figures 4(b)–4(d)). In the oim/oim mice, both SrR and Aln treatment induced a significant reduction in Tb.Sp (-32% SrR; -28% Aln) compared to the Veh treatment (Figure 4(e)). In the wt/wt mice, only SrR treatment significantly increased Tb.Th (+19%) as compared to the Veh group. Aln did not affect Tb.Th for either genotype (Figure 4(f)).

**Figure 5:**
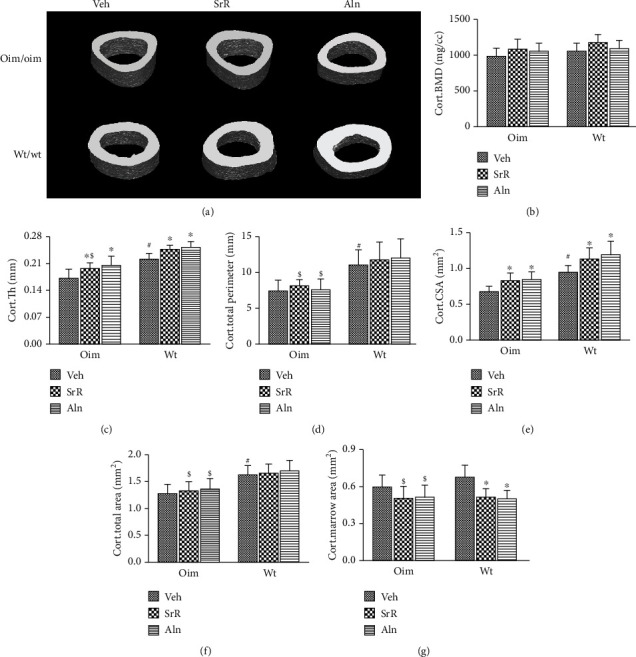
Effects of different treatments on femora cortical bone mass. (a) Representative micro-CT images of cortical regions in femora reveal positive effects on bone cortices with SrR or Aln treatment. (b) SrR or Aln treatment had no alteration in cortical BMD for both genotypes. (c, e) Cort.Th, and Cort.CSA were significantly increased with SrR or Aln in both wt/wt and oim/oim mice. (d, f) No significant changes were observed in total area and periosteal perimeter after SrR or Aln treatment for both genotypes. (g) Marrow area was significantly decreased after SrR or Aln treatment in wt/wt mice, while only a small decreasing trend was observed in oim/oim mice. Values were expressed as mean ± SD; *n* = 6 animals per group for oim/oim and wt/wt groups (missing data due to deformity or fractures). ∗*p* < 0.05, compared with the same genotype vehicle. ^#^*p* < 0.05, wt/Veh vs. oim/Veh. ^&^*p* < 0.05, SrR vs. Aln treatment for the same genotype. ^$^*p* < 0.05, wt/Veh vs. oim/SrR group or oim/Aln group.

At the femoral middiaphysis, no significant differences were found in cortical BMD among the genotypes within any of the treatment groups (Figure 5(b)). oim/Veh mice had thinner cortices compared to wt/Veh mice. Eleven weeks of SrR or Aln treatment significantly increased Cort.Th (+13% oim and +10% wt in SrR; +20% oim and +13% wt in Aln) and Cort.CSA (+23% oim and +19% wt in SrR; +25% oim and +26% wt in Aln) in both oim/oim and wt/wt mice (Figures 5(c) and 5(e)). Indeed, the Cort.Th and Cort.CSA in the Aln-treated group showed a trend of increase in both genotypes, although the differences failed to reach statistical significance (Figures 5(c) and 5(e)). SrR or Aln treatment had no alteration in total area and periosteal perimeter for both genotypes (Figures 5(d) and 5(f)). In contrast, the marrow area was significantly decreased after SrR or Aln treatment in wt/wt mice (-23% SrR; -26% Aln), while only a small decreasing trend was observed in oim/oim mice (-17% SrR; -14% Aln) (Figure 5(g)).

### 3.5. Bone Mechanical Properties

At the end of the treatment, the structural mechanical properties (moment of inertia, maximum load, stiffness, and energy to failure) and intrinsic material properties (ultimate stress and total strain) were greater in wt/Veh mice compared to oim/Veh mice (Table 1). Femora from oim/Veh mice showed a more brittle phenotype, reflected by lower postyield displacement, an inverse indicator of bone brittleness. Eleven weeks of SrR or Aln treatment significantly improved the structural properties including the moment of inertia (+47% oim and +25% wt in SrR; +58% oim and +37% wt in Aln), maximum load (+33% oim and +26% wt in SrR; +42% oim and +29% wt in Aln), and stiffness (+59% oim and +29% wt in SrR; +69% oim and +34% wt in Aln) in both genotypes. Both SrR- and Aln-treated oim groups showed increases in stiffness to levels not significantly different from the Vehicle-treated wt/wt mice. There were no significant differences in these three parameters between SrR- and Aln-treated mice. In addition, the energy to failure was significantly increased after SrR or Aln treatment in wt/wt mice (-70% SrR; -74% Aln), while only a small increasing trend was observed in oim/oim mice (-53% SrR; -64% Aln). The estimated material properties (Young's modulus, ultimate stress, and total strain) were not affected by SrR or Aln treatment in either wt/wt or oim/oim mice. Similarly, SrR or Aln treatment had no effects on yield force, yield displacement, postyield displacement, and total displacement (Table 1).

### 3.6. Bone Formation and Differentiation

**Figure 6:**
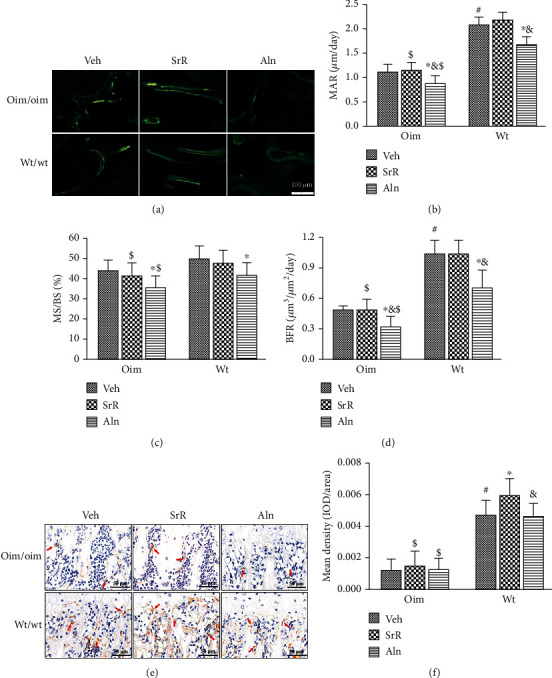
Effects of different treatments on bone formation and differentiation. (a) Tetracycline labels observed by fluorescence light microscopy in the frontal slices of the proximal tibia. (b–d) oim/Veh mice had lower bone formation than wt/Veh mice, as reflected by lower MAR and BFR. Bone formation parameters including (b) MAR, (c) MS/BS, and (d) BFR were significantly reduced by Aln treatment, while remained unchanged by SrR treatment. (e) Representative images of immunohistochemical staining for type I collagen expression. (f) Quantitative analyses showed that type I collagen expression was elevated in the wt/wt mice, with no alteration in the oim/oim mice. Alendronate had no effect on the expression of type I collagen in both genotypes. Values were expressed as mean ± SD; *n* = 6 animals per group in tetracycline labels and *n* = 8 animals per group in immunohistochemical staining (missing data due to fracture or deformity). ∗*p* < 0.05, compared with the same genotype Veh treatment. ^#^*p* < 0.05, wt/Veh vs. oim/Veh. ^&^*p* < 0.05, SrR vs. Aln for the same genotype. ^$^*p* < 0.05, wt/Veh vs. oim/SrR group or oim/Aln group.

Bone formation (MAR and BFR) was significantly lower in oim/Veh mice than in wt/Veh mice (Figure 6). In the SrR groups, bone formation was maintained in both genotypes shown by the absence of diminution of MAR and BFR after 11 weeks of treatment as compared with the Veh-treated groups (Figures 6(b) and 6(d)). In the Aln groups, however, all parameters of bone formation (MAR (-21% oim; -19% wt), MS/BS (-19% oim; -16% wt), and BFR (-35% oim; -31% wt)) significantly decreased after the treatment in both genotypes compared to the Veh-treated groups (Figures 6(b)–6(d)), reflecting a marked diminution of bone turnover.

Immunohistochemical staining examination showed that the level of Type I collagen in the Veh-treated wt/wt mice was significantly higher than that of the oim/oim mice, indicating that osteoblasts were more mature in wt/wt mice (Figures 6(e) and 6(f)) than in oim/oim mice. After 11 weeks of treatments, the expression of type I collagen was significantly upregulated in the wt/wt mice, while no alteration was observed in the oim/oim mice (Figures 6(e) and 6(f)). Alendronate had no effect on the expression of type I collagen in both genotypes.

### 3.7. Bone Resorption

**Figure 7:**
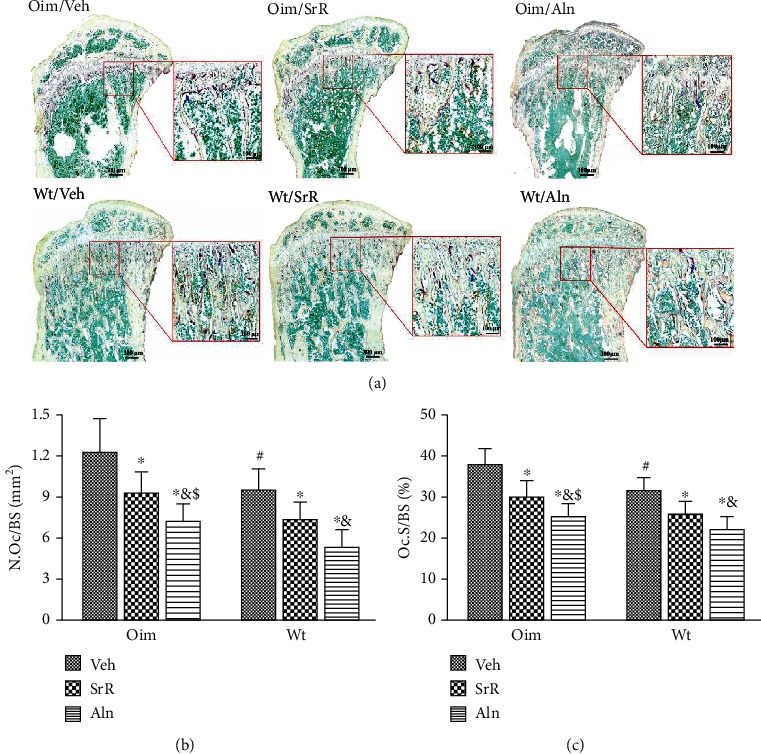
Effects of different treatments on bone resorption. (a) Representative images of TRAP staining in proximal tibia from different groups. (b, c) oim/Veh mice had higher bone resorption than wt/Veh mice, as reflected by higher osteoclast number (N.Oc/BS) and osteoclast surface (Oc.S/BS). (b) N.Oc/BS and (c) Oc.S/BS were significantly decreased with SrR or Aln treatment in both wt/wt and oim/oim mice. The N.Oc/BS and Oc.S/BS decreased to a greater extent with Aln treatment than with SrR treatment. Values were expressed as mean ± SD; *n* = 8 animals per group (missing data due to fracture or deformity). ∗*p* < 0.05, compared with the same genotype Veh treatment. ^#^*p* < 0.05, wt/Veh vs. oim/Veh. ^&^*p* < 0.05, SrR vs. Aln for the same genotype. ^$^*p* < 0.05, wt/Veh vs. oim/SrR group or oim/Aln group.

TRAP staining examination showed that bone resorption parameters (N.Oc/BS and Oc.S/BS) were significantly higher in oim/Veh mice than in wt/Veh mice (Figure 7). After 11 weeks of treatment, both SrR and Aln induced a significant decrease in Oc.S/BS (-20% oim and -18% wt in SrR; -33% oim and -30% wt in Aln) and N.Oc/BS (-24% oim and -23% wt in SrR; -42% oim and -44% wt in Aln) in either oim/oim or wt/wt mice, indicating a marked inhibition of bone resorption (Figures 7(b) and 7(c)). During the study period, bone resorption parameters decreased to a greater extent with Aln treatment than with SrR treatment (Figures 7(b) and 7(c)).

## 4. Discussion

Bisphosphonate has been the traditional, first-line drug to treat OI. Previous evidence suggests that SrR may be a potential alternative to treat OI [10]. However, the comparative effects of SrR and BP in OI treatment remain unknown. In this study, we compared the effect of SrR and Aln on fracture reduction in oim mice. Both SrR and Aln resulted in similar decreases in the fracture incidence and similar architectural and biomechanical changes in bone in a mouse model of OI. The effect of SrR on fracture reduction was through inhibiting bone resorption while maintaining bone formation. The effect of Aln, however, was mainly through inhibiting bone turnover.

Both SrR and Aln have been used to treat osteoporosis for decades [12, 33]. These two drugs can significantly reduce vertebral and/or nonvertebral fractures in osteoporotic patients and animals [12, 33–37]. In this study, we found that both SrR and Aln caused a significant reduction in fractures, from 4.2 fractures per mouse in Veh-treated oim mice to 2 fractures in SrR-treated mice and 1.8 fractures in Aln-treated mice. No significant difference was found between these two drugs. To explore possible mechanisms of the anti-fracture efficacy of these two agents, we first evaluated the bone architecture changes after the treatment. Consistent with previous studies evaluating the efficacy of either SrR or Aln alone [10, 23, 38], our study showed that both treatments significantly improved the femoral trabecular parameters (BMD, BVF, and Tb.N) and cortical parameters (Cort.Th and Cort.CSA). However, no significant differences were observed in these parameters between the two treatments. These findings are consistent with previous reports by Chen et al. [37] and Sun et al. [36]. Rizzoli et al. [24] treated 88 osteoporotic postmenopausal women with SrR or Aln for 2 years. It was found that SrR and Aln groups did not differ in BMD and Tb.N, similar to our findings. However, the SrR group had greater increases in distal tibia Cort.Th and Cort.CSA than the Aln group in their study [24]. In comparison, we found that the femoral Cort.Th and Cort.CSA between Aln and SrR treatment were not significantly different. This discrepancy may be explained by the different locations that were measured and the different pathogenesis of OI and postmenopausal osteoporosis. Interestingly, Tb.Th was only increased with SrR in wt/wt mice, while no change was found with Aln in either genotype. This finding was in line with a previous study which showed no significant change in Tb.Th with Aln treatment [23]. Our results indicated that SrR improved BVF by increasing Tb.N and Tb.Th, while Aln, an antiresorptive therapy, improved BVF only by Tb.N. Collectively, these findings suggest that SrR treatment may have a dual effect on bone mass, while Aln may mainly rely on antiresorption activity.

Bone strength is an indicator of changes in bone fragility after treatment. In this study, the maximum load and stiffness of the midshaft femur were improved with both SrR and Aln treatments in either genotype (without any difference between groups). This increase was associated with an improvement in the cortical bone mass. These findings were similar to previous studies in Aln- and/or SrR-treated OI mice [10, 23, 25] and osteoporotic rats [37, 39]. Chen et al. [37] compared the effect of SrR and Aln on femoral biomechanical properties. In their experiment, two biomechanical tests were applied at the femur diaphysis and femur metaphysis. No difference in the diaphysis bending test was observed between the two groups. However, in the metaphysis bending test, biomechanical parameters (maximum load, energy to failure, and stiffness) in the Aln group were significantly higher than those in the SrR group. In our study, we only performed the bending test at the femur diaphysis and found no significant difference between groups. Rizzoli et al. [24] established a finite element model to estimate the biomechanical parameters of the distal tibia. It was found that SrR significantly increased the energy to failure of the distal tibia compared with baseline, whereas Aln did not. Their finding was in contrast with ours, which may be explained by differences in the models and biomechanical tests used between studies.

Strontium ranelate is considered a dual-acting agent with both antiresorptive and anabolic skeletal benefits [15–17]. Aln is a powerful and well-tolerated bisphosphonate agent, which mainly suppresses osteoclast differentiation and activity [40]. In this study, both SrR and Aln treatment reduced the number of osteoclasts and osteoclast surface. Aln showed a greater effect than SrR, in line with the decreased serum level of NTx. In another study [41] conducted in 387 postmenopausal women with osteoporosis, it was found that Aln but not SrR decreased bone resorption parameters. This finding suggests that Aln has a stronger effect on the inhibition of bone resorption activity than SrR. Osteoclasts secrete factors that can affect osteoblasts. Thus, drugs inhibiting bone resorption could lead to a proportional inhibition of bone formation due to this coupling phenomenon [42]. In couple with its effects on bone resorption, Aln treatment significantly inhibited bone formation parameters (MAR, MS/BS, and BFR). In contrast, bone formation parameters were maintained rather than inhibited by SrR treatment in our study. Previous in vitro studies showed that SrR could increase preosteoblast replication and differentiation [15, 43] and decrease osteoclastogenesis and osteoclast-mediated bone resorption [15, 44]. The dual effect of SrR on bone metabolism has also been confirmed by in vivo animal studies [16, 17]. In monkeys and ovariectomized rats [16, 17], bone formation was maintained and bone resorption decreased by SrR, which was consistent with our findings. In glucocorticoid-induced osteoporotic rats, Sun et al. [36] demonstrated that SrR treatment prevented the decrease in bone formation (MS/BS, MAR, and BFR/BS), which was induced by glucocorticoid administration. In postmenopausal osteoporotic women, Chavassieux et al. [41] found that Aln had much stronger effects on the inhibition of bone formation than SrR. Collectively, both our study and these two comparable studies [36, 41] showed that SrR treatment resulted in a higher bone formation rate than Aln treatment. Although Aln had an inhibitory effect on bone formation as compared with SrR, the improvements in bone mass and bone strength are comparable between these two drugs. This finding could be explained by the much lower bone resorption in the Aln group than in the SrR group.

Long bone growth and overall weight gain were maintained with both agents. Our observation is consistent with Bargman et al. study [22], which showed that long bone growth was not affected by Aln treatment. However, other studies indicated that Aln may have inhibitory effects on long bone growth [45, 46]. This inconsistency may be attributed to the different dosages used between studies. Evans et al. [45] found significantly shortened humerus and ulna with a higher dosage of Aln treatment (2.5 mg/kg/wk), while no effect was observed with a lower dosage (0.125 mg/kg/wk). SrR has been reported to cause side effects on the cardiovascular system [47]. In this study, two mice were dead in the SrR group, which may not be attributed to the cardiovascular problems as two mice in the Aln group and one in the Veh group were also dead (no difference was found between these groups). These deaths might be related to genetic mutations of the model [46]. Therefore, these two agents are likely safe for the treatment of OI.

There are several limitations to this study. A major limitation was the small sample size. We found significantly reduced fracture incidence after SrR and Aln treatment. However, this study may be underpowered to detect group differences in several parameters such as mechanical tests. Future studies with a larger sample may help to shed more lights on the specific mechanisms for this phenomenon. Another limitation is that Sr^2+^ could directly and artificially increase BMD by incorporating into the bone matrix as a substitute for lighter calcium ions. The BMD data may have been confounded by this artifact. In addition, Faxitron was used to interpret the mechanics, which may not be as accurate as data from the micro-CT. There may be sex-dependent differences in response to pharmaceutical interventions for the treatment of OI. Only female mice were included in this study. Thus, findings may not be generalized to male nice. Based on the above limitations, future larger studies consisting of both male and female rats are needed to confirm findings from this study.

## 5. Conclusions

In conclusion, SrR and Aln treatment demonstrated similar effects on fracture reduction in oim mice. Both SrR (1800 mg/kg/day) and Aln (0.21 mg/kg/week) significantly increased femoral BMD, bone mass, and biomechanical properties. Compared with Aln, SrR had an extra effect on maintaining bone formation but a lower inhibitory effect on bone resorption. Therefore, in clinical practice, SrR may be considered an option for patients with OI when other medications are not suitable or have evident contraindications.

## Figures and Tables

**Table 1:** Biomechanical properties of femoral cortical bone after 11 weeks of treatment (mean ± SD).

Parameters	oim/Veh (*n* = 6)	oim/SrR (*n* = 6)	oim/Aln (*n* = 6)	wt/Veh (*n* = 6)	wt/SrR (*n* = 6)	wt/Aln (*n* = 6)
Moment of inertia (mm^4^)	0.074 ± 0.013	0.109 ± 0.019∗^$^	0.117 ± 0.022∗	0.134 ± 0.021^#^	0.168 ± 0.028∗	0.183 ± 0.021∗
Maximum load (N)	12.6 ± 1.6	16.7 ± 1.6∗^$^	17.8 ± 2.0∗^$^	21.6 ± 1.9^#^	27.2 ± 3.5∗	27.9 ± 3.2∗
Stiffness (N/mm)	82.9 ± 10.4	131.6 ± 33.7∗	140.2 ± 20.9∗	125.5 ± 44.3^#^	161.3 ± 30.3∗	168.5 ± 29.5∗
Energy to failure (mJ)	1.22 ± 0.18	1.85 ± 0.42	1.98 ± 0.46	3.93 ± 1.23^#^	6.70 ± 1.24∗	6.85 ± 1.48∗
Young's modulus (MPa)	5549 ± 1113	5027 ± 1007	5985 ± 996	5052 ± 750	5251 ± 1083	5698 ± 824
Total strain	0.035 ± 0.005	0.040 ± 0.005^$^	0.038 ± 0.009^$^	0.081 ± 0.023^#^	0.089 ± 0.026	0.084 ± 0.026
Ultimate stress (MPa)	112.8 ± 13.8	121.9 ± 15.5	127.5 ± 20.7	136.2 ± 19.3^#^	149.6 ± 15.4	155.0 ± 23.6
Yield force (N)	11.5 ± 2.4	13.9 ± 2.1	13.6 ± 2.1	16.6 ± 2.8^#^	18.7 ± 3.3	18.3 ± 3.3
Yield displacement (mm)	0.091 ± 0.024	0.094 ± 0.015	0.088 ± 0.020	0.090 ± 0.021	0.085 ± 0.025	0.087 ± 0.019
Postyield displacement (mm)	0.087 ± 0.022	0.119 ± 0.027^$^	0.094 ± 0.041^$^	0.233 ± 0.026^#^	0.264 ± 0.041	0.250 ± 0.047
Total displacement (mm)	0.178 ± 0.020	0.213 ± 0.027^$^	0.182 ± 0.035^$^	0.324 ± 0.034^#^	0.349 ± 0.049	0.338 ± 0.046

*Notes*. Veh, vehicle; SrR, strontium ranelate; Aln, alendronate. ∗*p* < 0.05, significantly different compared with same genotype vehicle. ^#^*p* < 0.05, significantly different between wt/Veh vs. oim/Veh. ^&^*p* < 0.05, significantly different between SrR vs. Aln treatment for the same genotype. ^$^*p* < 0.05, significantly different between wt/Veh vs. oim/SrR group or oim/Aln group.

## Data Availability

Original data and source files will be made available upon request from the corresponding authors, Dr. Ying Zhang and Dr. Hailong He.
